# Patients’ experiences with a behaviour change intervention to enhance physical activity in primary care: A mixed methods study

**DOI:** 10.1371/journal.pone.0212169

**Published:** 2019-02-12

**Authors:** Heleen Westland, Jill Sluiter, Sophie te Dorsthorst, Carin D. Schröder, Jaap C. A. Trappenburg, Sigrid C. J. M. Vervoort, Marieke J. Schuurmans

**Affiliations:** 1 Julius Center for Health Sciences and Primary Care, University Medical Center Utrecht, Utrecht, the Netherlands; 2 Center of Excellence in Rehabilitation Medicine, Brain Center Rudolf Magnus, University Medical Center Utrecht University Utrecht, Utrecht, the Netherlands; 3 De Hoogstraat Rehabilitation, Utrecht, the Netherlands; 4 Cancer Center, University Medical Center Utrecht, Utrecht, the Netherlands; 5 Education Center, UMC Utrecht Academy, University Medical Center Utrecht, Utrecht University, Utrecht, the Netherlands; Weill Cornell Medical College in Qatar, QATAR

## Abstract

**Objective:**

To explore the experiences of patients at risk for cardiovascular disease in primary care with the Activate intervention in relation to their success in increasing their physical activity.

**Methods:**

A convergent mixed methods study was conducted, parallel to a cluster-randomised controlled trial in primary care, using a questionnaire and semi-structured interviews. Questionnaires from 67 patients were analysed, and semi-structured interviews of 22 patients were thematically analysed. Experiences of patients who had objectively increased their physical activity (responders) were compared to those who had not (non-responders). Objective success was analysed in relation to self-perceived success.

**Results:**

The questionnaire and interview data corresponded, and no substantial differences among responders and non-responders emerged. Participating in the intervention increased patients’ awareness of their physical activity and their physical activity level. Key components of the intervention were the subsequent support of nurses with whom patients’ have a trustful relationship and the use of self-monitoring tools. Patients highly valued jointly setting goals, planning actions, receiving feedback and review on their goal attainment and jointly solving problems. Nurses’ support, the use of self-monitoring tools, and involving others incentivised patients to increase their physical activity. Internal circumstances and external circumstances challenged patients’ engagement in increasing and maintaining their physical activity.

**Conclusion:**

Patients experienced the Activate intervention as valuable to increase and maintain their physical activity, irrespective of their objective change in physical activity. The findings enable the understanding of the effectiveness of the intervention and implementation in primary care.

**Trial registration:**

ClinicalTrials.gov NCT02725203.

## Introduction

Cardiovascular disease is the leading cause of death worldwide, and the mortality rates are expected to increase in the next few decades [1]. It is well established that healthy behaviours, including physical activity, lower the risk of events related to cardiovascular disease, comorbidities, and mortality [2–4]. National guideline for the desirable levels of physical activity recommend at least 30 minutes of moderate to vigorous activity five days per week [5,6]. A majority of patients do not meet this target [7], underlining the need for effective strategies to promote physical activity. In the Netherlands, patients at risk for cardiovascular disease are monitored, treated and supported in primary care. In collaboration with the general practitioner, primary care nurses play a pivotal role in monitoring treatment outcomes and promoting healthy behaviour [8], However, nurses’ support to patients in adopting healthy behaviour is often brief and fragmented throughout the consultation [9–11], Structured behaviour change support using behaviour change techniques (BCTs), such as goal setting, action planning and self-monitoring is lacking in most consultations [11], To adequately support patients in changing their behaviour, nurses need to change their traditional consultation style towards a coaching-oriented consultation style [12–14]. This implies that in order to improve physical activity in patients, nurses also need to change their own behaviour. To enhance this behaviour change in patients and nurses the Activate intervention was developed using the Behaviour Change Wheel [15,16]. A behavioural analysis for both the behaviour of patients and the behaviour of nurses was made using the COM-B (capability, opportunity, motivation-behaviour) model [15,16]. The application of the Behaviour Change Wheel resulted in the development of the Activate intervention for patients and a standardised training programme for nurses, in which nurses were equipped with the required competences to deliver the intervention according to the protocol [16].

The effectiveness of the Activate intervention is currently being evaluated in a cluster-randomised controlled trial in adult patients at risk for cardiovascular disease in general practices in the Netherlands. To enhance our understanding of the effectiveness of the intervention and to explore how the intervention works in individual patients, a parallel process evaluation from the perspective of the patient alongside the Activate trial was conducted using both quantitative and qualitative research techniques [17–20]. Furthermore, insight into patients’ experiences with the intervention and the extent to which they perceive success in increasing their physical activity might enable our understanding of what might occur when implementing the intervention in routine practice [17,19,20]. Nurses’ perspectives regarding the delivery and feasibility of the Activate intervention are described elsewhere [21].

The aim of this study was to explore the experiences of patients at risk for cardiovascular disease in primary care with the Activate intervention in relation to their success with the intervention regarding increasing their physical activity.

## Methods

### Study design

A convergent mixed methods design nested within a cluster-randomised controlled trial was used to enhance the understanding of patients’ experiences with the Activate intervention [22]. Quantitative data were collected using a questionnaire and were converged with qualitative data from semi-structured interviews, which contained questions regarding the different components of the Activate trial and patients’ achieved results.

### The activate trial

Subsequent to this study, the Activate intervention is being evaluated in a two-armed cluster-randomised controlled trial in primary care in the Netherlands, comparing the Activate intervention with care as usual according to the Dutch guideline of cardiovascular risk management [6]. The Activate trial includes 31 participating general practices, 36 primary care nurses and 195 patients (Activate trial, ClinicalTrials.gov NCT02725203). A detailed description of the development and content of the intervention has been described elsewhere [16]. As a result of the behavioural analysis according to the Behaviour Change Wheel, the Activate intervention is structured around 17 BCTs, including goal setting, action planning, feedback on behaviour, review behavioural goals, problem-solving and self-monitoring. The intervention consists of four standardised nurse-led consultations to enhance physical activity, spread over a 12-week period: one consultation in the first week and the following consultations after 2, 6 and 12 weeks. Consultations occurred in the patients’ own general practice, with a duration of 20–30 minutes. Patients received a workbook, which included tips and tricks, useful websites, activity logs and action plans, and they were equipped with an accelerometer (personal activity monitor; Pam AM300) [23] in order to self-monitor their daily physical activity.

The analysis of what nurses need to change in order to adequately deliver the intervention to patients resulted in a selection of 21 BCTs, which are integrated into a standardised, comprehensive training programme for nurses. This training programme consists of a one-day knowledge and skills training in behaviour change theories and counselling, instructional videos on how to apply the BCTs in the consultations, a scripted handbook and checklists describing what to do when. Furthermore, nurses received two individual coaching sessions by a health psychologist. Prior to the training nurses were asked to watch two short videos: an instructional video of the study procedures and a video of background information on the medical concerns of physical activity regarding patients at risk for cardiovascular disease. The primary outcome is patients’ physical activity, measured with an accelerometer (personal activity monitor; Pam AM300)[23], and operationalised as the number of minutes of moderate (3–6 metabolic equivalents (METs)) to vigorous (≥6 METs) physical activity, with a 6-month follow up period (T2). The Pam AM300 is a small, valid, and reliable tri-axial accelerometer which patients were asked to wear on the hip for seven consecutive days for 12 hours daily. The Pam AM300 registers activity data of minutes a day in a sedentary category (<1.8 METs), a living category (1.8–3 METs), a moderate category (3–6 METs), and a vigorous category (>6METs), which is uploaded from a docking station.

Patient data are collected at baseline (T0), after completion of the intervention (T1) and three months after completion of the intervention (T2). Data collection comprised filling in a questionnaire and wearing the accelerometer for at least four weekdays and one weekend day for 8 hours. The activity information of the accelerometer was and remained blinded to patients to ascertain objectivity of the measurements, leaving patients unaware of their objective level of physical activity.

### Sampling and recruitment

#### Questionnaire

The study sample consisted of all patients (n = 93) from general practices (n = 16) situated throughout the Netherlands who participated in the Activate trial and were allocated to the intervention group. Patients were included in the analysis if they completed all four consultations, completed the questions about their experiences with the Activate intervention which were embedded in the T1 questionnaire and wore the accelerometer at T0 and T1. A total of 67 (72.0%) patients were included in the analysis. Patients who dropped out during the intervention (n = 18), omitted to complete the questionnaire (n = 7) or had invalid accelerometer data (n = 1) were excluded from the analysis. Patients dropped out during the intervention due to health concerns (n = 6), personal circumstances (n = 3), burden too high (n = 3), achieved satisfied level of physical activity (n = 3), and other reasons (n = 3).

#### Semi-structured interviews

From the 67 eligible patients, a sub-sample of 22 patients was purposively selected based on either being successful or not successful in increasing their physical activity. The increase was measured using patients’ objective change from baseline to 3 months of follow up (T1) for moderate to vigorous physical activity according to the accelerometer. Patients’ success of increasing their physical activity was defined as a mean difference in minutes of moderate and vigorous physical activity by at least 20% at T1 compared to baseline [16]. A total of 11 patients who succeeded in achieving this threshold (responders) and 11 patients who did not achieve this threshold (non-responders) were included in the study. Furthermore, patients were selected with a wide range regarding age, sex, educational level, and living situation to maximise the diversity of patients in the sample. Selected patients were invited by an invitational letter to participate in the study. To respond, patients could contact the researchers. Patients who did not respond were contacted by telephone within one week to inquire whether they would like to participate in the study and, if desired, were provided with additional information. When patients were willing to participate in the study, an appointment was scheduled. If patients refused, they were asked whether they would like to give a reason for refusal. If so, patient data and the reason for refusal were reported. New patients were purposively selected from the research database to replace them. Purposive sampling was used until the maximum variation in the sample and data saturation were achieved. In total, 29 patients were invited to participate, and 22 patients (75.9%) distributed over 11 general practices agreed to participate. Patients refused to participate due to time constraints (n = 1) or personal circumstances (n = 1), or they did not report a reason (n = 5).

### Data collection

#### Questionnaire

Patients’ experiences were explored by a post-intervention questionnaire, which they received directly after they completed the intervention between June 2016 and April 2017. The questionnaire was developed by three members of the research team, and face validity was assessed by the research team and two additional researchers who are experts in conducting process evaluations of complex interventions. Questions regarding patients’ perceptions of the intervention, their success of increasing their physical activity and their motivation and confidence towards maintaining their physical activity levels were measured on a five-point Likert scale, ranging from 1 (strongly disagree) to 5 (strongly agree). Questions regarding helpful components of the intervention to increase their activity levels were measured on a four-point Likert scale, ranging from 1 (strongly disagree) to 4 (strongly agree). Additionally, patients were asked to report the components that were most helpful to them in order to increase their physical activity. Characteristics of patients were collected at the start of the Activate intervention.

#### Semi-structured interviews

Semi-structured individual telephone interviews were performed to evaluate patients’ experiences with their participation in the Activate intervention and perceived success with regards to their physical activity. An interview guide with open questions was used. The topics addressed in this guide regarded patients’ experiences with the intervention, their expectations of their participation, perceived success and maintenance of their physical activity, their experiences with the different components (including the most prominent BCTs) of the intervention and their satisfaction with the intervention (S1 Appendix). All interviews started with the same question: “What was the reason you agreed to participate in the Activate study?”

The interviews were conducted by a nurse scientist in training and a medical doctor in training (JS, SD) who were familiar with having contact with patients and were not involved in the intervention or in other aspects of care for the participating patients. Patients were unknown to the interviewers, and patients were interviewed once at the patients’ preferred time and date. The mean duration of the interviews was 30.30 minutes (range: 22.04–40.31 minutes). All interviews were audio-recorded. During and directly after the interviews, memos were made regarding observations, reflections on methodological issues, initial thoughts related to emerging themes, and refinements of the interview guide. Before the study interview training was provided to the interviewers by an expert on qualitative research (SV) and a nurse scientist (HW). During the study, the interviewing techniques of the interviewers were discussed by members of the research team (SV, HW). The interviews were conducted between November 2016 and March 2017.

### Ethics

The Activate trial, including this process analysis, was ethically reviewed and approved by the Medical Ethics Research Committee of the University Medical Center Utrecht (NL54286.041.15). All patients gave written informed consent prior to the start of the Activate trial. Prior to the interviews, informed consent was obtained verbally. This study was conducted in accordance with the Declaration of Helsinki.

### Data analysis

#### Questionnaire

Data were analysed and presented according to the patients’ level of success (responder or non-responder), defined as a mean difference in minutes of moderate and vigorous physical activity according to the accelerometer by at least 20% at T1 compared to baseline [16]. Patient characteristics and the most helpful components of the Activate intervention as perceived by the patients were presented as numbers and corresponding percentages. Patients’ perceptions towards the intervention, their success of the intervention and their motivation and confidence to maintain of Activate intervention were presented as a median and interquartile range (IQR). Quantitative data were descriptively analysed using the Statistical Package for the Social Sciences (SPSS version 21; Chicago, IL, USA).

#### Semi-structured interviews

Qualitative data were analysed according to the six phases of thematic analysis of Braun and Clarke using a realist method [24]. Data analysis started after the first four interviews. In phase 1 (familiarizing with the data), the interviews were transcribed verbatim (JS, SD), and after every four interviews, the transcripts were checked for accuracy, read to get an overall picture and re-read to grasp the details (JS, SD, HW). During this phase, initial ideas for coding were discussed (HW, JS, SD, SV). In phase 2 (generating initial codes), transcripts were systematically and independently coded and discussed in the research team after every four interviews (HW, JS, SD, SV). In phase 3 (searching for themes), the research team collated codes into meaningful themes whose relevance emerged from several interviews. A preliminary description of potential themes and subthemes was made and discussed (HW, JS, SD, SV). In phase 4 (reviewing themes), potential themes were reviewed for consistency with the transcripts to ensure the validity of the themes with the entire data. Potential themes were further refined (HW, JS, SV). In phase 5 (defining and naming themes), the specific content of each theme was further worked out using the transcripts, and themes were named and defined (HW, JS, SV). In phase 6 (producing the report), the report was drafted, and vivid quotes to illustrate the themes were selected (HW, JS, SV) and reviewed (HW, JS, SD, JT, CS, SV, MS). Data saturation was achieved prior to completing the 22 interviews; however, as planned, the interviews were continued to ensure a maximum variation in the sample of responders and non-responders. Data analysis was supported by NVivo 11.0 software (QSR International Pty Ltd, Version 11.0, 2011).

To increase the credibility of the data, the validity of the data was ensured by researcher triangulation and peer review throughout the phases of the study [25]. An expert on qualitative research (SV) was involved in all phases of the data collection and data analysis to further strengthen the accuracy and dependability of the process [25]. The process of data analysis was systematically discussed by the research team (HW, JS, SD, SV). The study’s conformability was ensured by an audit trail [25]. The use of the 15-point checklist of Braun and Clarke [24] confirmed the correct application of the six phases of thematic analysis; see S2 Appendix. The 32-point consolidated criteria for reporting qualitative studies (COREQ) was used to facilitate reporting of the results [26]; see S3 Appendix. Memo writing and expert opinion were used to support the analysis and to enhance study reliability [27].

## Results

### Questionnaire

Patients’ characteristics are reported in Table 1. The results of the questionnaires are presented in Tables 2 and 3. Patients’ characteristics were generally similar across both responders and non-responders (Table 1), except for employment, level of education, and physical activity (Table 1).

**Table 1:** Characteristics of patients.

Characteristics		Questionnaire (n = 67)		Interview (n = 22)	
		Responder^a^ (n = 25)	Non-responder^a^ (n = 42)	Responder^a^ (n = 11)	Non-responder^a^ (n = 11)
Female, n (%)		11 (44.0)	17 (40.5)	7 (63.6)	5 (45.5)
Age in years, mean ± SD		62.6 ± 7.8	61.6 ± 9.5	61.8 ± 7.7	61.7 ± 11.7
Employed n (%)		8 (32.0)	17 (40.5)	4 (36.4)	5 (45.5)
Living with others, n (%)		22 (88.0)	35 (83.3)	9 (81.8)	9 (81.8)
Native Dutch, n (%)		24 (96.0)	41 (97.6)	10 (90.9)	11 (100)
Level of education, n (%)					
	Primary education or below	2 (8.0)	NA	2 (18.2)	NA
	Secondary education	16 (64.0)	34 (81.0)	7 (63.6)	10 (90.9)
	Higher education	6 (24.0)	8 (19.0)	2 (18.2)	1 (9.1)
	Unknown	1 (4.0)	NA	NA	NA
Physical activity^**a**^					
	Living, baseline, mean ± SD	99.1 ± 31.4	108.8 ± 35.6	112.4 ± 34.3	103.1 ± 26.3
	Living, 3 months, mean ± SD	96.3 ± 30.7	104.0 ± 41.8	97.8 ± 29.6	101.7 ± 34.6
	Moderate, baseline, mean ± SD	34.4 ± 16.3	42.3 ± 20.8	42.5 ± 15.5	48.4 ± 19.0
	Moderate, 3 months, mean ± SD	49.7 ± 20.8	37.6 ± 18.3	58.5 ± 22.3	43.3 ± 17.6
	Vigorous, baseline, mean ± SD	0.6 ± 0.8	1.8 ± 4.3	0.5 ± 0.4	1.8 ± 2.8
	Vigorous, 3 months, mean ± SD	1.5 ± 1.9	0.7 ± 1.4	1.3 ± 1.7	1.1 ± 1.8

^**a**^According to the accelerometer. Data is divided in categories: living category: 1.8–3 METs; moderate category: 3–6 METs and vigorous category: >6METs

NA: not applicable

**Table 2:** Patients’ experiences with the effectiveness of the Activate intervention on their physical activity.

Statements	Total n = 67	
	Responder^a^ (n = 25) Median [IQR]	Non-responder^a^ (n = 42) Median [IQR]
My physical activity increased in the last 3 months^b^	4 [2]	4 [1]
I am satisfied with my level of physical activity^b^	4 [1]	3.5 [1]
I perceive my present level of physical activity as pleasant^b^	4 [1]	4 [1]
I am motivated to maintain my level of physical activity^b^	4 [1]	4 [0]
I feel confident to maintain my level of physical activity^b^	4 [1]	4 [1]
I intend to maintain my level of physical activity^b^	4 [1]	5 [1]
Participating in the Activate intervention helped me to increase my physical activity^c^	3 [1]	3 [0]
Generally, I perceived the support during the Activate intervention as pleasant^c^	3 [1]	3 [1]
The consultations with the nurse helped me to increase my physical activity^c^	3 [1]	3 [0]
Wearing the accelerometer helped me to increase to increase my physical activity^c^	4 [1]	3 [1]
Keeping the activity log helped me to increase my physical activity^c^	3 [1]	3 [1]

^a^According to the accelerometer data

^b^ measured on a five-point Likert scale: 1 (strongly disagree) to 5 (strongly agree)

^c^ measured on a four-point Likert scale: 1 (strongly disagree) to 4 (strongly agree)

*Abbreviations*: IQR interquartile range

**Table 3:** Characteristics of interview participants.

ID	Male/ Female	Age	Living alone	Level of education	Change in minutes of moderate to vigorous physical activity from baseline		
					According to patient	According to accelerometer Mean diff. minutes (%)	
R1	Male	74	Alone	Secondary education	o	Non-responder	- 15.4 (-44.8%)
R2	Male	73	Not alone	Primary or below	+	Non-responder	- 6 (-12.8%)
R3	Female	69	Not alone	Secondary education	+	Responder	+ 27.9 (+48.4%)
R4	Male	65	Not alone	Secondary education	o	Non-responder	- 3.7 (-10.9%)
R5	Female	68	Alone	Secondary education	+/-	Responder	+10.3 (+21.7%)
R6	Female	57	Not alone	Secondary education	+	Non-responder	- 12.1 (-17.6%)
R7	Female	53	Not alone	Higher education	o	Responder	+ 40 (+81.4%)
R8	Male	70	Not alone	Primary or below	+	Responder	+ 9.4 (+21.7%)
R9	Female	40	Alone	Higher education	o	Non-responder	- 16.7 (-21.8%)
R10	Male	71	Not alone	Secondary education	+	Non-responder	- 0.4 (-4.2%)
R11	Male	66	Not alone	Secondary education	+	Responder	+21.7 (+35.9%)
R12	Female	68	Not alone	Secondary education	o	Responder	+10 (+44.6%)
R13	Male	49	Not alone	Secondary education	+	Non-responder	- 7.4 (-12.5%)
R14	Female	49	Alone	Secondary education	o	Responder	+ 8.3 (+23.6%)
R15	Female	71	Not alone	Secondary education	+	Non-responder	+ 7.4 (+17.3%)
R16	Male	63	Not alone	Higher education	+	Responder	+ 14.0 (+116.7%)
R17	Female	48	Not alone	Secondary education	o	Non-responder	+ 1.0 (1.4%)
R18	Female	63	Not alone	Primary or below	+	Responder	+ 20.9 (+32.8%)
R19	Female	62	Not alone	Secondary education	+	Non-responder	+ 2.3 (+4.8%)
R20	Male	50	Not alone	Secondary education	+	Responder	+ 10.6 (+25.0%)
R21	Female	61	Not alone	Secondary education	+	Responder	+ 11.0 (+27.5%)
R22	Male	69	Not alone	Secondary education	o	Non-responder	- 12.9 (-21.3%)

+ physical activity increased; +/- physical activity increased a little; o physical activity did not increase much, but participation increased health or awareness of the impact of physical activity on their health

Responders were on average less physically active at baseline compared to non-responders. However, at three months of follow-up, responders substantially increased their average number of minutes in the moderate to vigorous category compared to non-responders who became less active on average.

**Fig 1:**
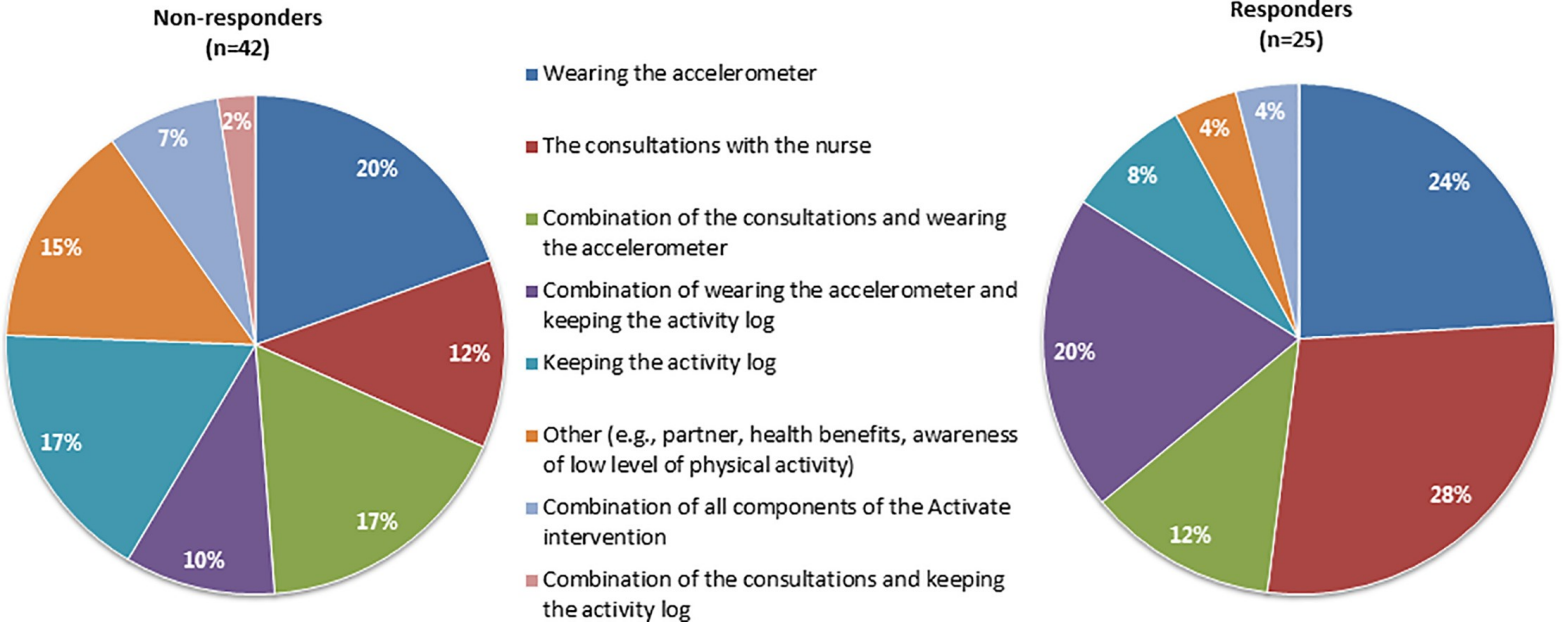
Patients’ perceived most helpful components of the Activate intervention to increase their physical activity.

Moreover, patients’ experiences with the effectiveness of the Activate intervention on their physical activity were also generally similar across both groups (Table 2). Generally, patients experienced an increase in their physical activity during the intervention period as a result of the intervention. Most patients were satisfied with their achieved results. Overall, patients were motivated, felt confident, and intended to maintain their achieved results. Generally, patients were pleased with the nurse-led consultations, wearing the accelerometer and keeping the activity log. Differences between responders and non-responders were apparent in the perceived most helpful components of the intervention; see Fig 1. Responders perceived the consultations with the nurse (28.0%), wearing the accelerometer (24.0%) and the use of both self-monitoring tools (20.0%) as the most helpful components for increasing their physical activity. Non-responders perceived the combination of the consultations and wearing the accelerometer (17.1%), keeping the log (17.1%) and other components, such as having a supporting partner and perceiving health benefits due to their participation in the intervention (14.6%), as being most helpful.

### Semi-structured interviews

A total of 22 patients (11 responders and 11 non-responders) were interviewed. Seven patients in the responder group were female compared to five women in the non-responder group. Overall, maximum variation regarding age, sex, educational level and the living situation was achieved; see Tables 1 and 3. All patients perceived an increase in their physical activity compared to baseline; however, to different extents. Thirteen patients felt they increased their physical activity (seven responders and six non-responders). Eight patients felt that their physical activity did not increase much, but their participation increased their health or awareness of physical activity on their health (three responders and five non-responders). The perceptions of nine patients (three responders and six non-responders) did not correspond with their objective measured success; see Table 3.

Generally, there was a substantial overlap between the experiences of patients with the intervention in both groups. Therefore, the themes were drawn from patients’ experiences as a whole, unless the data showed a substantial distinction between both groups, which is reported accordingly. The overview of the themes that emerged from the analysis is included at S4 Appendix.

Patients’ engagement with becoming more active: All patients were aware that being physically active would positively affect their health. Patients often reported their intention to increase their physical activity; however, they could not achieve this increase on their own. Nurses’ requests to participate in the intervention aligned with this intention, and the impact of physical activity on their health additionally prompted them to participate. Furthermore, some patients specifically wanted insight into their current amount of physical activity, which prompted them to participate in the intervention. Due to their perceived needs, most patients’ felt highly engaged to participate in the intervention and confident in their ability to increase their physical activity. A small difference was seen between responders and non-responders. At the start of the intervention, responders tended to be less motivated compared to non-responders, and they more often reported physical or emotional constraints of becoming more physically active. Despite these constraints, these patients felt confident to increase their activity because of positive beliefs about the intervention and the support of their nurse. Once patients consented to participate in the intervention, they felt committed to their consent.

*“I felt that I should be more active, and this intervention came at the right time*. *I thought that I had to take advantage of it…I could not succeed in that myself. And then I thought ‘Well, this is a nice opportunity to see if I will succeed with this support.’ …It is not something I would have picked up myself.” (R14, responder)*

*“I was really ready for it, I just wanted to start it and when I start something …I'm not going to say, ‘I don’t feel like doing it or something.’ Then, I should not participate*.*” (R6, non-responder)*

Perceived effects of becoming more active: Most patients felt satisfied with their achieved results. Achieving positive effects resulted in a higher engagement with the intervention and maintenance of increased activity. Patients perceived both physical effects, such as feeling fitter and needing less medication, and emotional effects, such as experiencing better mood and becoming more socially active. Disappointing results negatively affected patients’ engagement with the intervention. All patients reported an increase in their physical activity and incorporated this into their daily lives, but to different extents. Patients who reported major improvements were likely to be responders and felt highly engaged in their achieved results. Some patients felt overjoyed by their achieved results, as their results exceeded their prior expectations, which highly motivated them to maintain their increased physical activity. Patients who reported minimal improvement in their physical activity were more likely to be non-responders.

*“At some point*, *I want to see* results. *If I don’t get a result in spite of all my efforts* … *then I stop the effort because it is meaningless* … *Then*, *the motivation is gone immediately; at least it is immediately affected*.*” (R22*, *non-responder)*

*“I also enjoy doing it … I chat with every owner of a dog. It is much more easy-going…Yes, that was very different from when I started. Then, I'd cower and never say anything…Well, I was shy and not feeling so good… Now*, *I even appeared in the diabetes newspaper …I would never have done that before.” (R21, responder)*

Increased awareness through participating in the intervention: At the start of the intervention, all patients were aware of the positive effect of physical activity on their health, and patients did not feel the need to read additional information. Despite their prior knowledge, the focus on physical activity in the intervention increased patients’ awareness of the importance of being active and its relation to their health. This raised awareness prompted patients to be active daily.

*“Of course, I knew that physical activity was important …but I'm much more aware now, certainly. And yes, if I have done nothing at all during a day, eh*, *I think ‘Yes, that’s actually not so wise.’ Let’s walk or do something then. So, it’s always on my mind.” (R4, non-responder)*

Regardless of their perceived extent of increased physical activity, all patients became aware of the amount and intensity of their physical activity, which they highly valued and which positively affected their engagement in the intervention and their ability to maintain activity. Their awareness was particularly raised by wearing the accelerometer and keeping the activity log. Additionally, nurses’ feedback on their level of goal attainment, reviewing their set goals and action planning also increased their awareness.

*“In the past, I had no idea; I thought, ‘I was walking with the dog,’ and besides that, I did not know actually…Honestly, I was not aware of it; it was not something that was on my mind … Yes*, *for me that was important, certainly.” (R6, non-responder)*

Perceived trustful relationship with the nurses: All patients knew the nurses from their prior routine consultations. Patients highly valued their trustful relationship with the nurse, in which they felt that they could share their honest thoughts without being judged. Their relationship with the nurse prompted patients to participate in the intervention. Patients’ perceived their relationship as crucial to increase their physical activity, as nurses’ feedback and review of their level of goal attainment offered them an incentive to attain their goals. Some patients reported that they were highly engaged to attain their goal, since they did not want to disappoint the nurse. Patients often felt rewarded by their nurses’ approbation of their attained results. Although some patients felt pressured or controlled by the nurse, they experienced that nurses’ support stimulated them to attain their goals.

*“During the intervention, she consciously involved me very much … She was very enthusiastic and friendly, and she did not judge … I could be honest … When it did not work out, for example, she did not get angry about that or anything. That was just very pleasant*.*” (R14, responder)*

*“Just because of our conversations … I had an incentive. Because I want to show that I have done something. And I don’t think ‘Well, next week then’ … so, it worked for me that there is someone who looks at and discusses what I have done. Well, that went all in a pleasant way. Yes*, *I think that helped me.” (R15, non-responder)*

Valuing nurses’ focus on increasing physical activity: Patients’ highly valued the subsequent focus on physical activity during the consultations. Almost all patients reported that, in particular, setting specific and attainable goals, combined with planning their actions, directed them towards increasing their physical activity. The agreed upon goals stimulated their commitment to attain those goals. Patients highly valued nurses’ feedback and review of their level of goal attainment, which positively affected their engagement in attaining their goals.

*“I liked that …because you know what you have to do and what your goal is. I am someone who likes to work towards a goal, that stimulates me …Somehow, I know, that’s what I’m doing it for*, *that’s what I want to accomplish.” (R6, non-responder)*

*“You’ve set your goal, and between the second or the third or the fourth consultation, you know what gets tough …Well, then, I went to the nurse, and she said, ‘Just try again’. I benefitted quite a lot from her support. Yes, because she immediately asked me ‘How did it go’? I said ‘Well, not exactly the way I wanted it to be.’ Then I could talk about it with her, which made me think, ‘Well, guys*, *I'll just continue.”(R19, non-responder)*

Almost all patients experienced nurses’ support in jointly setting specific and attainable goals as very helpful, as without this support they tended to set general, unrealistic or unchallenging goals. A few patients reported that setting goals and planning actions did not match with their unstructured personality or personal circumstances, and therefore, they did not value these elements. These patients perceived this advice as unhelpful and sought their own activities.

*“It is difficult for me to see what is realistic and what’s not. How do you start with something … I found it difficult to make it more realistic and especially in more bite-sized chunks, in a way that I could oversee it. By clearly indicating, ‘Are you not going a bit too fast, you want too much, and is it not more convenient to be active within smaller bouts, which are much more feasible and lead to more results instead of disappointing yourself?’ That certainly helped me*. *She made me realise that I did not have to run a marathon immediately. That was nice.” (R14, responder)*

*“Yes, she provided me with ideas …but that did not really work for me. For example, that I could walk to do the shopping; however, that costs me too much time! Then*, *I jumped on the bike again.” (R7, responder)*

Involving others to increase physical activity: Although a few patients preferred solitary activities, most patients experienced that getting support from their family and friends engaged them to improve their physical activity. For some patients, involving others was a prerequisite for improving their physical activity. Family members and friends were seen as common facilitators to be physically active. In particular, spouses who joined the patient in increasing their physical activity often engaged patients to attain their goals.

*“And I also do it together with my husband, and yes, I think it's just great that he joined me … We also encourage each other…I like doing that together, and by doing it together, I’m even more motivated …if you're busy doing things at home, one of the two says, 'Hey! Shall we go for a walk now?’ Then*, *we stop our activities and go for a walk.” (R3, responder)*

Despite the fact that patients enjoyed being active with others, patients felt demotivated when they had to decrease their activity speed to match others. Being active with someone who had an equal or higher walking speed challenged them to increase and maintain their physical activity.

*“All those people walk a lot slower than me …Well, I can walk with someone like that, but then I have to adjust my walking speed. I also walk with my wife sometimes …but she doesn’t walk as fast as I do*. *Then, you are busy adjusting your walking speed instead of having a nice walk …” (R22, non-responder)*

Physical activity was also seen as an opportunity to meet new people and extend their social contacts, which increased their enjoyment in being physically active and prompted them to maintain being physically active.

*“Because I enjoy everything, and I am really eager to go to the gym …I have a lot of confidence in the people who teach there. I really don’t want to let it go anymore. It's just those people together, afterwards, we drink coffee with each other … you get new contacts … Well*, *I think that's great too.” (R21, responder)*

Furthermore, being active with others often involved making a commitment, which was often regarded as an incentive to being active. The accountability towards others strengthened their engagement.

*“It’s at a fixed time. So, if I want to go*, *then I have to be there…and if I walk on my own, then I sometimes think, 'I really don’t feel like going or I'll do it later.' That kind of thing. Then, I postpone it, and in this case, I can’t.” (R18, responder)*

Insight into physical activity using self-monitoring tools:Generally, patients regarded the use of the self-monitoring tools such as the accelerometer and activity log as very helpful and stimulating to increase their physical activity because they provide insights into their amount and intensity of physical activity. This insight often challenged patients to extend their activity to attain their goals and to compete with prior results.

*“Uh, I will not say it's a game to put a number on the activity log, but it's just that I’m looking somewhere halfway through the day and think, ‘Well, it’s okay to walk a bit further this evening.’ …It's nice to monitor myself and to see where I actually win and where I lose something on my schedule*. *I can just browse back and review the results of last week. So, yes, I think it's helpful to estimate a little how to pick it up or adjust it again.” (R13, non-responder)*

Despite being highly valued by most patients, some patients reported that failing to attain their goals or not trusting the accuracy of the accelerometer demotivated them to increase their physical activity.

*“Sometimes I thought, ‘Well, I just wanted to have done this much’ …And, of course, I did not succeed every day, even if I sometimes felt that I had done quite a bit …And then I thought, ‘No*, *I have not done enough’ …Then, I felt a bit down …I had the feeling that I did a lot or very much …and then I thought, ‘Well it is just disappointing’.” (R19, non-responder)*

Patients differed in how they perceived the need to use the tools. Some patients reported that once they were aware of the amount and intensity of their physical activity, using the tools was no longer necessary, whereas others continued to use the tools because they felt the need to be stimulated to be physically active, and they perceived the tools as an incentive.

*“No*, *I don’t need an activity meter anymore because I know now when I walk that round, it’s a one-hour walk.” (R11, responder)*

The majority of patients said they wore the accelerometer and kept the log daily. Most patients registered their activity at the end of the day, while others registered their activity directly afterwards or when it suited them best. Keeping the log prompted them to reflect on their level of goal attainment, which raised patients’ awareness of their physical activity. All patients reported the time spent wearing the accelerometer, and keeping the log was acceptable to them.

*“In the evening after dinner, I thought, ‘Well, I'll just sit down on the couch. I do not have to walk anymore …so, I can take off the activity meter.’ That was the moment to fill in the activity log …I like to do that kind of thing to get insight into what happened, ‘What did I do and what conclusions can I draw from that?’ Yes, I liked it …sometimes, I felt like ‘I had done too little, I have to walk, I have to move’ …So, yes that surely helped me …Writing down, monitoring myself, looking back to what I did last week*. *I thought that was great.” (R14, responder)*

Most patients found the accelerometer and log easy to use; however, some patients reported technical and practical problems while using the tools. A few patients lost the accelerometer or lost their activity data because the accelerometer automatically resets after midnight. Losing their activity data made them feel disappointed, as they had to estimate their activity levels, which prompted them to find ways to prevent losing the data in the future.

*“Well*, *look, it's not annoying to wear that thing. You put it in your pocket and it measures, so …it does not bother me, or I do not forget about it. The only thing that is awkward is, I think, at midnight, it resets itself. I have had a few times that I lost my measures from that day.” (R13, non-responder)*

Taking responsibility to increase their physical activity: Despite patients feeling stimulated by both the nurses’ support and the self-monitoring tools, patients often reported that in the end, they themselves are responsible for increasing their physical activity and for maintaining their health.

*“You start something, and then you keep track of certain goals …You have your own responsibility for something that you promise to do. That you have to be corrected a bit sometimes, well that is logical*, *and that’s what happened …You are actually constantly thrown back onto yourself, ‘You have to do it and …there is no one else who is going to do that.” (R11, responder)*

Patients believed that taking responsibility for their health also included being honest with themselves and the nurses about their level of goal attainment. Not being honest was perceived as useless for themselves and the nurses.

*“We are both open to each other …if you keep something back, then it’s of no use going there …then, you’re fooling yourself …That is a waste of time for both*; *bothering someone who is serious.” (R17, non-responder)*

Perceiving the need to use reminders:The majority of patients did not use the reminders they received at the start of the intervention to be active, such as post-it notes and a pen with the study logo. Patients often felt reminded by the self-monitoring tools and by storing the log in a visible place. Other patients did not feel the need to use reminders as they felt self-motivated to be active or were reminded by their spouses.

*“I didn’t use those post-its and pen, no. That log helped me …and we are each other's support …Yes, we are each other's stimulus.” (R3*, *responder)*

Physical capability impacts becoming more active:A majority of patients reported having (chronic) physical constraints, such as asthma, back pain, or joint aches. Some patients had existing physical constraints prior to their participation in the study, while others mentioned a health problem occurring during the intervention. Having physical constraints negatively affected patients’ self-confidence in achieving their desired results, as they often had to reduce their goals and felt hampered in planning activities and finding tailored activities. This often affected their engagement and made them feel negative about themselves and their participation in the intervention. Patients often perceived difficulties in finding alternatives for being active despite their physical constraints. Patients valued nurses’ support in jointly seeking for alternatives, such as finding suitable activities and adapting their activity speed to their circumstances. They often felt strengthened by this support, which helped them persevere to attain their goals.

*“I have bursitis in the shoulder, and now I have a tennis elbow, so every time something happens, you know…that makes me think, ‘How annoying.’ I want to do more but it doesn’t work, I just can’t…I think that’s so unfortunate. Then, I have to boost myself and just try, and if it doesn’t work, then it doesn’t work…. Still, it is mainly thinking, ‘I'm just going to try it, and if I don’t succeed*, *then I have bad luck and I'll only cycle a small lap.” (R19, non-responder)*

Continually dealing with circumstances affecting being physically active:The majority of patients reported internal and external circumstances that affected their ability to increase and maintain being active. Perceived internal circumstances included enjoying being active and having physical constraints. Perceived external circumstances included weather and season, patients’ working environment, busy family lives, being abroad, cancelation of their activity buddy and taking care of a sick family member. Despite patients’ willingness to being physically active, these circumstances challenged patients in prioritising daily physical activity. Furthermore, patients felt challenged in finding ways to address these circumstances themselves. Almost all patients valued nurses’ support in jointly finding alternatives and in tailoring activities to patients’ preferences and personal circumstances. Finding ways to address their circumstances helped patients to create routines and to persevere in being physically active in daily life. Most patients were able to address their circumstances by adapting their thoughts by focussing on the range of possibilities instead of the limitations, such as incorporating multiple short bouts of physical activity into each day or purchasing home exercise equipment. Patients perceived that being physically active despite their hampering circumstances strengthened their engagement and confidence to maintain their activity after the intervention.

*“If the weather was very bad …Then, I did some extra cycling on the home trainer. That is what I discussed with the nurse, that’s what we agreed on. When you don’t actually go outside, then I'm still moving*.*” (R10, non-responder)*

*“I have actually noticed that*, *despite the fact that I want to move more, having dogs, young children, and a busy job, I find it quite difficult to pick a moment to be really active …Well, what I have done more often is bringing my children to school by bike instead of taking the car.” (R13, non-responder)*

Intending to maintain being physically active after the intervention: After finishing the intervention, all patients intended to maintain being physically active. However, patients felt that, in particular, ceasing their incentives, such as nurses’ support, wearing the accelerometer and keeping the log, challenged them in maintaining their achieved level of physical activity. Patients who succeeded in building their activities into their daily lives felt confident in maintaining their achieved level of physical activity.

*“When you are doing your usual things again, then yes, you have to think about it carefully, you're less aware, compared to when you're really in that process …Of course, it is now that I know a little bit, if I walk that far or do that much, how much that is. I didn’t know that before, so now I know that bit just by heart …But because you do not have to go back to the nurse anymore, then you think, ‘Well, no one knows about it …except yourself …Yes, that check, that seems to be necessary*.*” (R6, non-responder)*

*“It doesn’t cost me a lot of extra effort. That’s especially after my work, I say ‘It’s a matter of incorporating it into my routine*.*” (R9, non-responder)*

## Discussion

### Principal findings

Patients who participated in the Activate intervention were satisfied with the intervention. The results from both the questionnaires and the interviews showed that the Activate intervention led to an increased awareness in patients of the importance of physical activity for their health and an increased awareness of the amount and intensity of their current physical activity. Irrespective of their objective changes in activity levels, patients perceived that they became more active and that they benefitted both physically and emotionally from their participation. Getting support from the nurses with whom they have a trustful relationship, including goal setting, action planning, feedback, and reviewing goals, as well as self-monitoring their amount and intensity of activity and involving others, were perceived as highly supportive and incentivised patients to increase and maintain their physical activity. Patients felt responsible for attaining their goals and honestly reflected on their achieved results with themselves and the nurses. Patients perceived that the self-monitoring tools prompted them to be active, and therefore, they did not feel the need to use other reminders. Furthermore, patients’ ability to increase and maintain being active was continually challenged by internal circumstances, such as enjoyment and physical constraints, and by external circumstances, such as weather and lack of time.

### Comparison with other studies

Patients felt they increased their physical activity due to the intervention. However, patients’ perceptions towards their success in many cases did not accurately align with their objective measured success. This is also seen in studies of the evaluation of experiences of older adults with the large PACE-UP and PACE-Lift trials, aiming at increasing patients’ level of moderate to vigorous physical activity by a walking intervention [28,29]. Some patients underestimated their objective success, while some patients overestimated their objective success. Both groups were broadly similar in their characteristics and experiences with the Activate intervention, although non-responders were more physically active at baseline compared to responders. This might be explained by the fact that patients with a relatively high baseline physical activity level might easily reach a ceiling level and suggest the complexity of correctly estimating one’s the amount and intensity of their physical activity. Responders tended to be less motivated to increase their physical activity level and reported more often physical and emotional constraints. It might have been that the physical activity level of these patients showed more room for improvement.

Patients perceived physical and emotional benefits of their increased physical activity, which positively affected their engagement in increasing and maintaining their activity; this has also been found in other studies [28,30]. Patients’ increased awareness also engaged them to continue and maintain being physically active [28–31].

The importance of involving others in initiating and maintaining physical activity has been widely reported [28,30,32]. Our study showed that family members and friends were facilitators, and in particular, spouses who joined the patient, which concurs with other studies [28,29,33,34]. Being active with others also positively affected patients’ enjoyment. Enjoying being active strongly engaged the initiation and maintenance of their physical activity, which aligns with other studies [28,35,36]. Additionally, in accordance with other studies, we also found that physical capability is important in initiating and maintaining physical activity [28,30,32,37,38]. Patients reported the need for having an incentive prompting them to be physically active, such as consenting to participate in the intervention, nurses’ subsequent support, wearing the accelerometer and keeping the log. Most incentives ceased after the intervention, and it remains uncertain whether and how the patients maintain being physically active. A study by Wahlich et al.[30] evaluated the maintenance of physical activity in mid-life and older adults after three years of follow up and reported that the facilitators, which helped to maintain regular activity, included maintaining good health, self-motivation, social support and good weather. These facilitators were also reported in our study, in which patients also received nurses’ support in finding alternatives to maintaining being physically active despite circumstances such as bad weather. In the study of Wahlich et al.,[30] patients’ lack of time was seen as the most important barrier to maintain being physically active. This is in line with our study, implying the importance of focussing on both initiating and maintaining behaviour change, such as finding ways to address circumstances and other conflicting goals or behaviours, which might increase the likelihood of maintaining being physically active [39,40].

Despite the fact that using prompts and cues has been shown to be effective to change behaviour [41], the majority of patients did not need additional prompts and cues to use the self-monitoring tools because they felt sufficiently motivated [30]. Consistent with other studies, patients highly valued the use of self-monitoring tools, facilitating them to increase their activity level [28,29,31]. However, patients reported that once they were aware of their amount and intensity or that the novelty of wearing the accelerometer had worn off, they no longer used the accelerometer, which is in line with other studies [30,42]. Furthermore, technical problems affected their engagement, which has also been reported [28,31]. Patients frequently reported the importance of having a trusting relationship with their nurse as being crucial for their participation in the intervention, as well as for their goal attainment, and its being an incentive, which aligns with other studies [34,43]. Despite patients highly valuing the accelerometer and the log, patients found nurses’ support invaluable in order to increase their physical activity, which has also been reported [44–47]. The subsequent consultations in which patients’ goals were reviewed and (re)set, feedback was received, and actions were planned were highly valued by almost all patients, as these consultations incentivised them to continue.

Furthermore, self-monitoring tools seemed inevitable in an intervention to increase activity, as patients highly valued having insight into the amount and intensity of their activity. This increased their awareness, and patients felt challenged and incentivised by using these tools. Additionally, self-monitoring is a likely effective BCT [48]. Van der Weegen et al., [44] found that the combination of nurse-led consultations with a self-monitoring tool was effective in increasing physical activity in primary care patients, whereas a solely counselling intervention by nurses was not effective when compared to routine care. This implies that interventions focussing on increasing physical activity need to include both the support of a trustful healthcare provider and self-monitoring tools.

### Strengths

The main strength of this study was the use of a convergent mixed methods design, wherein the triangulation of both quantitative and qualitative data was used to gain an in-depth exploration and understanding of patients’ experiences with the Activate intervention and their perceived success opposed to their objectively measured success.

Furthermore, this study was conducted alongside a cluster-randomised controlled trial, before the trial results are known, which prevents interpretation bias of the study results and enhances the understanding of the effectiveness once the results of the Activate trial are known.

To enhance dependability of the qualitative data, the interviewers were unknown to the patients prior to the interviews, inviting them to be more candid. During the entire process, data were independently analysed by three researchers and an independent expert in qualitative research. The trustworthiness was enhanced by an audit trail, memo writing, the use of Braun and Clarke’s checklist [24] and the COREQ [26].

### Limitations

The focus on moderate and vigorous physical activity in the maintained Dutch norm of physical activity and the applied threshold of 20% change in physical activity (responder versus non-responder) might underestimate the fact that low volumes of physical activity and small changes in physical activity also lead to health benefits [2–4].

After the intervention period, the initial study sample was reduced from 93 to 67 questionnaires and valid accelerometer datasets. Data collection for this study was embedded in the data collection for the Activate trial, and patients who dropped out of the intervention were also excluded from this study. These patients might have expressed different experiences, which could have affected the results. Furthermore, the interviews were conducted by telephone due to logistical reasons. Face-to-face interviews might have invited patients to elaborate on their answers more fully, which might have further enriched the quality of the data [49].

### Implications

Based on the insight gained into patients’ experiences with the Activate intervention and their perceived success, we have defined three recommendations that should be addressed in patients’ behaviour change support. First, interventions aiming to increase patients’ level of physical activity should include both self-monitoring tools and consultations with a healthcare provider who has a trustful relationship with the patient. Second, the effectiveness of such interventions can be enhanced by including the following BCTs: goal setting [41,50,51], action planning [52], reviewing behavioural goal(s) [41,51], feedback on behaviour [41,53], problem-solving [51,54], self-monitoring of activity [48,51], and involving others [41,51]. These BCTs were highly valued by patients and are most likely to be effective. Third, support focussing on dealing with both internal and external circumstances to increase patients’ physical activity in daily life is needed.

## Conclusion

Patients who participated in the Activate intervention were satisfied with the intervention. Patients experienced an increase in their awareness of the importance of physical activity for their health and an increase in their level of physical activity. Responders and non-responders did not differ substantially in their experiences with the intervention and their perceived success. Patients’ perceptions towards their success did not always align with their objective change in activity. Patients’ engagement in the intervention was affected by perceived physical and emotional benefits, level of goal attainment, and perceived incentives. Patients experienced the combination of self-monitoring tools and being supported by the nurses with whom they have a trustful relationship as being invaluable to increasing their physical activity. This mixed methods study has increased our understanding of patients’ experiences of their participation in a behaviour change intervention in primary care. The findings contribute to the evaluation of the effectiveness of the Activate trial and might facilitate implementation of such interventions in primary care.

## Supporting information

S1 Appendix Interview guide. (DOCX)

S2 Appendix Checklist of criteria for good thematic analysis: 15-point checklist. (DOCX)

S3 Appendix Consolidated criteria for reporting qualitative studies (COREQ): 32-item checklist. (DOCX)

S4 Appendix Overview of the potential themes, subthemes and key themes that emerged from the analysis. (DOCX)
