# Supplementary material for: Patients’ experiences with a behaviour change intervention to enhance physical activity in primary care: A mixed methods study
Source: PLoS One. 2019 Feb 12;14(2):e0212169. doi: 10.1371/journal.pone.0212169 (PMC6372184; doi:10.1371/journal.pone.0212169)
Supplement: S1 Appendix — (DOCX) [file pone.0212169.s001.docx]

# S1 Appendix Interview guide

| **Topics** | **Questions (examples)** |
| --- | --- |
| General | Standard opening question: What was the reason you agreed to participate in the Activate study? |
| Expectations prior to the study | What expectations did you have prior to the Activate study? Can you tell me to what extent your expectations were met? |
| Perceived outcome | Can you tell me whether you think you became more physically active due to your participation in the study? |
| Perceptions towards maintaining physical activity | Have you made any changes to your routine of life as a result of your participation in the study?  To what extend do you think you will maintain being physically active?  Now you have finished the intervention, how motivated are you to continue being more physically active? Now you have finished the intervention, how self-confident are you to continue being more physically active? How do you plan to maintain the changes you have made? |
| Perceived motivation towards increasing physical activity | At the start of the study, how motivated were you to increase your physical activity (grade 1-10)? Did your motivation change during the intervention period? |
| Perceived self-confidence towards increasing physical activity | At the start of the study, how self-confident were you to increase your physical activity (grade 1-10)? Did your self-confidence change during the intervention period? |
| Experiences with nurses’ support | Can you tell me how you experienced the nurses’ support during the consultations?  Can you tell me whether you think the consultations helped you increase your physical activity? |
| Perceptions towards the consultation structure and most prevalent BCTs | How did you experience to set personal goals and action plans? Did the nurses help you to set your own goals and plan your actions? [BCTs: goal setting and action planning] To what extent did this affect your progress? What is your opinion about reviewing the extent to which you attained your goals? [BCT: reviewing behavioural goal(s)] Did this affect your progress? Did the nurse discuss how you can get any support from e.g., family or friends? [BCT: social support] To what extent did this affect your progress?  Did you discuss strategies to maintain being physically active? Did you bring up the strategies yourself? [BCTs: habit formation, problem-solving and relapse prevention] Can you tell me how the nurse supported you if you were found it difficult to maintain your progress? What did you find helpful and unhelpful? [BCT: problem-solving]  Did the nurse prompt you to use any reminders to help you increasing your physical activity (e.g., the Activate study post-its, pen, etc.)? [BCT: prompts and cues] Did you find this helpful?  Can you tell me whether you think the study consultations differed compared to the routine consultations?  Did the nurse clearly explain to you what you were expected to do at home? (think about your goals, action plan, wearing the accelerometer, keeping the logbook) |
| Experiences with the study materials and equipment | Can you tell me to what extent wearing the accelerometer and keeping the activity logbook helped you to increase your physical activity? How did this affect your progress? When did you wear the accelerometer and keep the log? Did you perceive any difficulties while wearing the accelerometer or keeping the activity log? How did you handle this? At the start of the study, you received a workbook. Did you use this workbook? What is your opinion about the content of the workbook? |
| Most and least effective components | Can you tell me what you found most helpful in becoming more physically active?  Can you tell me what you found least helpful in becoming more physically active? |
| Duration of the intervention | What is your opinion about the number and length of the consultations? Generally, how much time did you spend on keeping the activity log? Was this acceptable to you? |
| Satisfaction with the intervention | Can you tell me how satisfied you are with your participation in the Activate study (grade 1-10)? Would you recommend this intervention to other patients? |
| Additional questions regarding previous or not discussed topics | Do you have anything to add to the questions I have asked? |
