# Supplementary material for: Patients’ experiences with a behaviour change intervention to enhance physical activity in primary care: A mixed methods study
Source: PLoS One. 2019 Feb 12;14(2):e0212169. doi: 10.1371/journal.pone.0212169 (PMC6372184; doi:10.1371/journal.pone.0212169)
Supplement: S2 Appendix — (DOCX) [file pone.0212169.s002.docx]

**S2 Appendix. Checklist of Criteria for Good Thematic Analysis: 15-point checklist**

Adapted from: Braun, V., & Clarke, V. Using thematic analysis in psychology. Qualitative research in psychology.2006; 3(2), 77-101.

| **Process** | **Criteria** | **Comment** |
| --- | --- | --- |
| Transcription | 1. The data have been transcribed to an appropriate level of detail, and the transcripts have been checked against the tapes for ‘accuracy’. | Reported in manuscript |
| Coding | 2. Each data item has been given equal attention in the coding process. | Yes |
|  | 3. Themes have not been generated from a few vivid examples (an anecdotal approach), but instead the coding process has been thorough, inclusive and comprehensive. | Reported in manuscript |
|  | 4. All relevant extracts for all each theme have been collated. | Reported in manuscript |
|  | 5. Themes have been checked against each other and back to the original data set. | Reported in manuscript |
|  | 6. Themes are internally coherent, consistent, and distinctive. | Reported in manuscript |
| Analysis | 7. Data have been analysed - interpreted, made sense of - rather than just paraphrased or described. | Reported in manuscript |
|  | 8. Analysis and data match each other – the extracts illustrate the analytic claims. | Reported in manuscript |
|  | 9. Analysis tells a convincing and well-organised story about the data and topic. | Reported in manuscript |
|  | 10. A good balance between analytic narrative and illustrative extracts is provided. | Reported in manuscript |
| Overall | 11. Enough time has been allocated to complete all phases of the analysis adequately, without rushing a phase or giving it a once-over-lightly. | Yes |
| Written  report | 12. The assumptions about, and specific approach to, thematic analysis are clearly explicated. | Reported in manuscript |
|  | 13. There is a good fit between what you claim you do, and what you show you have done – i.e., described method and reported analysis are consistent. | Reported in manuscript |
|  | 14. The language and concepts used in the report are consistent with the epistemological position of the analysis. | Reported in manuscript |
|  | 15. The researcher is positioned as *active* in the research process; themes do not just ‘emerge’. | Reported in manuscript |
