# Supplementary material for: Patients’ experiences with a behaviour change intervention to enhance physical activity in primary care: A mixed methods study
Source: PLoS One. 2019 Feb 12;14(2):e0212169. doi: 10.1371/journal.pone.0212169 (PMC6372184; doi:10.1371/journal.pone.0212169)
Supplement: S4 Appendix — (DOCX) [file pone.0212169.s004.docx]

**S4 Appendix. Overview of the potential themes, subthemes and key themes that emerged from the analysis**

| **Potential themes** |  | **Subthemes** |  | **Key themes** |
| --- | --- | --- | --- | --- |
|  |  |  |  |  |
| - Health benefits - Awareness of amount of physical activity - Intentions - Inability to change - Need for incentives - Trust in nurse - Confidence in own ability - Motivation to change - Commitment |  | - Perceiving the importance of physical activity on health - Raising awareness of amount of physical activity - Changing own behaviour does not work - Needing incentives and support to change - Trust in the nurse prompted to participate - Being confident to increase activity level - Having physical and emotional constraints influences motivation - Committing themselves after consent |  | Patients’ engagement with becoming more active |
|  |  |  |  |  |
| - Satisfaction - Success - Physical effects: more energy, feeling fitter, medication reduction - Emotional effects: better mood, more socially active - Daily routines |  | - Being satisfied with achieved results - Experiencing success influences engagement - Experiencing physical and emotional effects - Incorporating physical activity into daily life |  | Perceived effects of becoming more active |
|  |  |  |  |  |
| - Awareness of health benefits - Awareness of amount of physical activity - Engagement - Maintaining physically active - Awareness by self-monitoring tools - Awareness by nurses |  | - Improving awareness of physical activity effect on health - Gaining awareness of the amount of physical activity |  | Increased awareness through participating in the intervention |

| - Knowing the nurse - Trust - Relationship - Honesty - No judgements - Vulnerability - Openness - Incentive - Not wanting to disappoint the nurse - Rewarded |  | - Having a trustful relationship without being judged - Perceiving the relationship and support as an incentive for physical activity |  | Perceived trustful relationship with the nurses |
| --- | --- | --- | --- | --- |
|  |  |  |  |  |
| - Focus on physical activity - Goal setting - Action planning - Goal attainment - Feedback - Review on goal attainment - Commitment - Support as helpful - Alignment of support on personal circumstances |  | - Being stimulated to attain goals by subsequent focus on physical activity - Valuing nurses’ support - Being committed to goal attainment |  | Valuing nurses’ focus on increasing physical activity |

| - Solitary activities - Involvement of family and friends - Motivation - Matching activity speed - Social contacts - Commitment - Incentive |  | - Preferring solitary activities - Involving family and friends - Matching activity speed with others stimulates - Expanding and extending social contacts - Involving others implies commitment and incentivises |  | Involving others to increase physical activity |
| --- | --- | --- | --- | --- |
|  |  |  |  |  |
| - Self-monitoring - Self-competition - Goal attainment - Equipment used for monitoring - Trust - User-friendliness: easy to use, losing data, losing the accelerometer |  | - Using self-monitoring tools to improve and maintain physical activity - Having trust in the self-monitoring tools - Perceived user-friendliness of the self-monitoring tools |  | Insight into physical activity using self-monitoring tools |
|  |  |  |  |  |
| - Own responsibility - Taking responsibility - Honestly - Commitment |  | - Feeling responsible for increasing and maintaining physical activity - Taking responsibility involves being honest |  | Taking responsibility to increase their physical activity |
|  |  |  |  |  |
| - Received reminders at the start of the intervention - Self-monitoring tools - Self-motivation - Spouses |  | - Need for using reminders - Using self-monitoring tools, self-motivation and souses were used |  | Perceiving the need to use reminders |
|  |  |  |  |  |
| - Physical constraints - Confidence - Engagement - Tailored activities - Seek for alternatives |  | - Perceiving physical constraints affects patients’ confidence - Getting support in seeking for alternatives to patients’ personal circumstances |  | Physical capability impacts becoming more active |
|  |  |  |  |  |
| - Personal and contextual circumstances: enjoyment, physical constraints, weather, season, working environment, busy family life, being abroad, availability of a buddy, taking care of important others - Circumstances challenge goal attainment - Address circumstances |  | - Internal and external circumstances influencing patients’ ability to being and maintaining physically active - Getting support in finding alternatives and addressing circumstances |  | Continually dealing with circumstances affecting being physical active |
|  |  |  |  |  |
| - Intentions - Challenges - Changed daily life - Confidence |  | - Intending to maintain being physically active - Being challenged by ceased incentives |  | Intending to maintain being physically active after the intervention |
